# Preface of Special Issue "Molecular Therapies for Inherited Retinal Diseases"

**DOI:** 10.3390/genes11020169

**Published:** 2020-02-05

**Authors:** Rob W.J. Collin, Alejandro Garanto

**Affiliations:** Department of Human Genetics and Donders Institute for Brain, Cognition and Behaviour, Radboud University Medical Center, P.O. Box 9101, 6500 HB Nijmegen, The Netherlands

Inherited retinal diseases (IRDs) are a group of progressive disorders that lead to severe visual impairment or even complete blindness. IRDs display a vast heterogeneity, clinically as well as genetically, with over 250 genes identified in which mutations can cause one or more clinical subtypes of IRD. Long considered incurable diseases, intense research over the last two decades, combined with major technological advancements, have enabled the development of the first therapeutic approaches for these diseases. The approval of LuxturnaTM (voretigene neparvovec), a gene augmentation therapy vector for RPE65-associated IRD, by the US Food and Drug Administration and the European Medicines Agency, is considered a true milestone in the field, and has led to the development of similar, or different therapeutic strategies for many other subtypes of IRD. Despite these major achievements, there are still many aspects that can—and need to—be improved, including more insights into the relationship between genetic variation and cellular dysfunction, optimization of the vectors and sequences used, improving delivery methods, as well as understanding and modulating the (local) immune response. In addition, the extreme rarity of some genetic subtypes of IRDs poses an enormous challenge on the development of novel therapies, in terms of e.g., costs and regulatory affairs.

In this Special Issue of Genes, we focus our attention on molecular therapeutic approaches for IRD, i.e., strategies that aim to overcome the primary genetic defect, or its consequences, by using genetic material or small compounds to restore molecular and cellular function. The issue is comprised of original research articles as well as (mini-)reviews, on topics such as gene augmentation, RNA-based therapies, genome editing, proteostasis, small molecule approaches and delivery vectors. The manuscripts mainly contain preclinical research, varying from work in cellular systems to in vivo studies. Some reviews summarize the current stage of ongoing clinical trials, i.e., for CHM- and RPGR-associated IRD. We close this Special Issue with a contribution of the Foundation Fighting Blindness USA on the patient’s (organizations’) perspective on the current landscape, as well as a future perspective on the era that lies ahead of us. With this, we aim to provide a contemporary overview on the development and implementation of novel (personalized) therapies for IRD, and identify the tremendous possibilities as well as the key bottlenecks that currently exist.

